# Research on Coal Mine Building Compliance Inspection System Based on Accident Causation and BIM in China

**DOI:** 10.3390/ijerph192416466

**Published:** 2022-12-08

**Authors:** Xinchun Li, Xiaolin Zhang, Quanlong Liu, Yueqian Zhang, Xiao Gu, Zunxiang Qiu

**Affiliations:** School of Economics and Management, China University of Mining and Technology, Xuzhou 221116, China

**Keywords:** coal mine safety construction, BIM, Word2Vec, compliance check, accident causation

## Abstract

Coal mine construction projects have high risks, and non-compliant designs generated in the design stage will have adverse effects on subsequent construction and production stages. Therefore, it is of great importance to conduct effective preconstruction compliance inspections on coal mine construction designs. To make the compliance check of coal mine building design more rapid and effective, and to reduce the risks arising from the design phase, this study built a compliance inspection system for coal mine building design from the causes of coal mine accidents, using the Word2Vec word similarity calculation method and BIM platform secondary development technology. The system was tested and was found to be able to detect a 92.82% non-compliant component rate where the correct inspection rate was 97.68%. In addition, the inspection time for a single component was only 0.23 s. The construction of the compliance inspection system based on accident causes has changed the extensive inspection mode in the traditional manual model inspection, and the inspection no longer depends on the experience of inspectors, thus improving the efficiency and accuracy of coal mine building model inspection. The inspection focuses on the building elements with high risks, which achieves the purpose of risk control in the design stage.

## 1. Introduction

As mineral resource mining depths deepen, the difficulty of mine construction continues to increase [1]. Coal mine construction is affected by six factors: humans, machines, environment, technology, management, and information [2,3], so there are many unpredictable risk factors [4]. Non-compliant mine construction project design defects will bury potential safety hazards in subsequent construction and production processes [5]. In 2009, there were only two coal mine accidents caused by non-compliant design in China. Recently, the number of accidents caused by problems at the design stage is increasing in China. In 2019, there were 32 such accidents, which contributed to serious environmental and social impacts. For example, in May 2021, a large roof accident occurred in the outer section excavation face of the 104 transportation lane of a mine operated by the Xin’an Coal Industry of the Zaozhuang Mining Group due to an unreasonable temporary support design, which resulted in three deaths and one minor injury. Checking the engineering project drawings or models in the design stage can determine whether there are non-compliant design defects in the design of an engineering project that may lead to safety accidents [2]. Therefore, it is of great significance in reducing the occurrence of coal mine safety accidents to ensure the compliance and rationality of the design by conducting compliance inspections during the design stage of coal mine construction projects.

In previous studies, compliance inspections on drawings or models were usually manual comparison inspections. The difficulty of inspection is that the model data information and related specification information are complicated. When the model volume is large, there will be omissions in the inspection, so this paper introduces Building Information Modeling (BIM). The US National Building Information Modeling Standard defines BIM as a model digitization technology based on interoperability and open standards, which can realize the visualization of the building design process and has the characteristics of simulation, coordination, and system [6]. Previously, many scholars used BIM for risk management in the architectural design and construction process. On the one hand, a BIM is a platform that integrates planning and design, construction organization, operation management, and maintenance management. It runs through the whole process of an engineering project and can be used as a complete system for risk management. For example, Malekitabar et al. [7] used a BIM to identify five groups of safety risk drivers that can be detected in the design stage for risk identification. Zhang et al. [8] built an algorithm to automatically analyze building models and detect common fall hazards during building construction. Zhong et al. [9] proposed an ontology semantic framework under a BIM environment for the detection of the building environment. On the other hand, a BIM provides a data interface, which can be combined with database technology, remote sensing technology, and virtual technology for security management. For instance, Li et al. [10] realized an automatic safety risk identification based on a BIM platform by establishing a risk knowledge base and identifying the relationship between engineering information and risk knowledge. At present, the technical fields that BIMs can be used for are broad. Afzal and Shafiq [11] combined a 4D BIM with VR visualization to improve the staff’s ability to identify and respond to risks by improving the flow of information in a multilingual environment. Getuli et al. [12] applied BIM and VR technology to safety training in construction, providing a new way to build safety culture. With the application of a building information model (BIM), the process of storing, collecting, and processing building information resources is simplified [13]. In recent years, research on rule checking based on BIM models has gradually increased [14,15,16,17]. Fenves [18] used decision tables for the first time for the automatic checking of building steel structure design compliance. Pauwels et al. [19] constructed an inspection environment for the automatic inspection of building performance using semantic rules based on industry foundation classes. Luo et al. [20] developed a BIM-based compliance inspection system for deep foundation pit construction projects, which improved the efficiency and accuracy of rule inspections. With research developing, the accuracy of automated inspection has continuously improved. For example, Christoph et al. [21] used a digital BIM design process to create an evaluation model and to evaluate building interior specifications. According to the reviewed literature, BIM is an instrumental tool for risk inspection and management. Therefore, the research on BIM-based architectural design compliance inspection is very important for improving the quality of inspection work and the safety level of construction projects.

In addition, the traversal inspection method takes a long time. The recognition of the focus of inspections and the improvement of inspection efficiency is an urgent problem to be solved. The Word2Vec model was selected in this study to objectively and effectively determine the inspection focus. The Word2Vec model is a simple, open-source, two-layer word vector training tool released by Google. By mapping words to high-dimensional space, we can analyze the semantics of words at a deeper level and then measure the similarity between different words [22]. In recent years, Word2Vec has been widely used by scholars in the exploration of accident causes and security risks in text data. For example, Jing et al. [23] used Word2Vec to extract word vectors which were used to build a bidirectional long short-term memory (LSTM) model and obtain accident precursors of conventional chemical accidents such as explosions, fires, and poisoning. Huang et al. [24] proposed an automatic detection and tracking algorithm for sudden hot spots using traditional algorithms based on Word2Vec and a keyword similarity matrix to reduce the impact of negative events. Zhang et al. [25] used Word2Vec to analyze the comments of e-commerce platforms and then identified the hidden dangers of the rapid development of e-commerce. Moreover, Tan et al. [26] mined the relationship between various risks based on Word2Vec to identify the key risk factors for control operations. An et al. [27] combined Word2Vec with related clustering techniques to analyze the change law and evolution direction of Weibo topic influence, helping counterterrorism departments predict potential risks. In previous research, Word2Vec was mostly applied to the discovery of qualitative accident causation, which aimed to explain the mechanisms of accident occurrences and provide a basis for risk assessment but was not specific to quantitative descriptions of building components. Consequently, this paper uses the Word2Vec tool to find the focus of the inspection from the existing accident facts.

**Figure 1:**
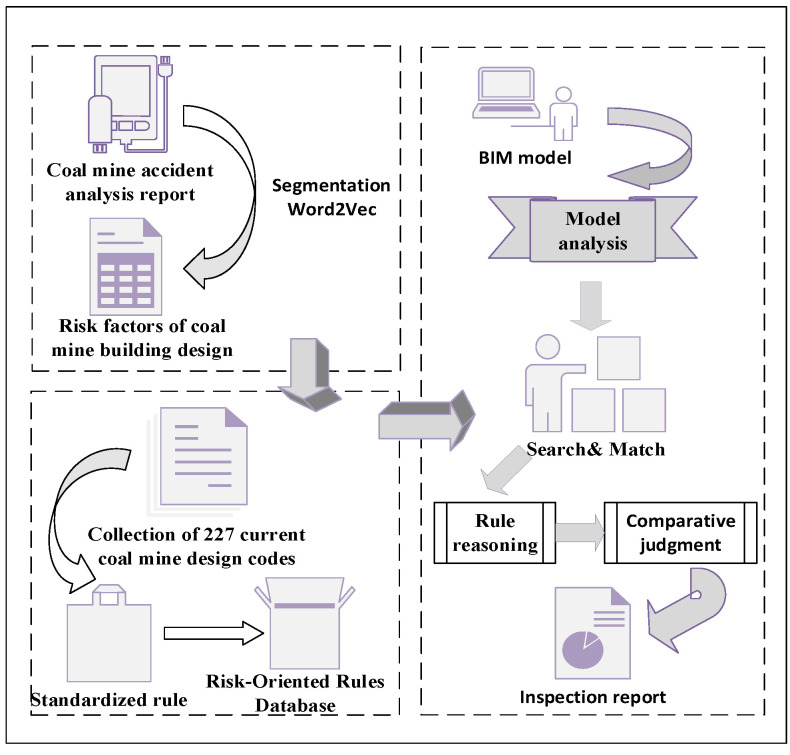
Research framework of this paper.

This study combines the Word2Vec word similarity calculation model and BIM to construct a compliance inspection system for coal mine building design. As shown in Figure 1, the research process of this paper includes the following steps. Firstly, use the Word2Vec tool to excavate the accident analysis report. By calculating the similarity between a certain type of accident word vector and the word vector of coal mine building components, the most relevant non-standard coal mine building elements in previous safety accidents are obtained. Secondly, the specifications of the current coal construction industry standards that constrain the construction elements extracted in the previous step are organized. On this basis, a database of standardized SQL rules that facilitates the next step of inspection is established. Finally, the secondary development of a BIM is carried out to link the SQL database and BIM to develop a compliance inspection system for the non-compliance inspection of high-risk building elements. By establishing a reasoning mechanism, the retrieval and matching of models and rules are realized for the purpose of checking non-compliant designs, which in turn enables the pre-control of safety hazards caused by the construction phase.

## 2. Extraction of Risk Factors for Coal Mine Building Design

### 2.1. Data Sources

China is rich in mineral resources and has a long history of mining, therefore its data volume is large and filterable. In addition, the construction standard system of China’s coal industry is relatively complete, which is convenient for citation in the subsequent construction process of the compliance inspection system. This paper collects the analysis reports of coal mine safety accidents that occurred in China from 2009 to 2021, and subsequently obtains 225 analysis reports of coal mine safety accidents caused by coal mine architectural design problems after data cleaning. These accident analysis reports include information such as accident overview, number of casualties, property damage, and accident cause analysis, which can be used as original data for text analysis.

**Figure 2:**
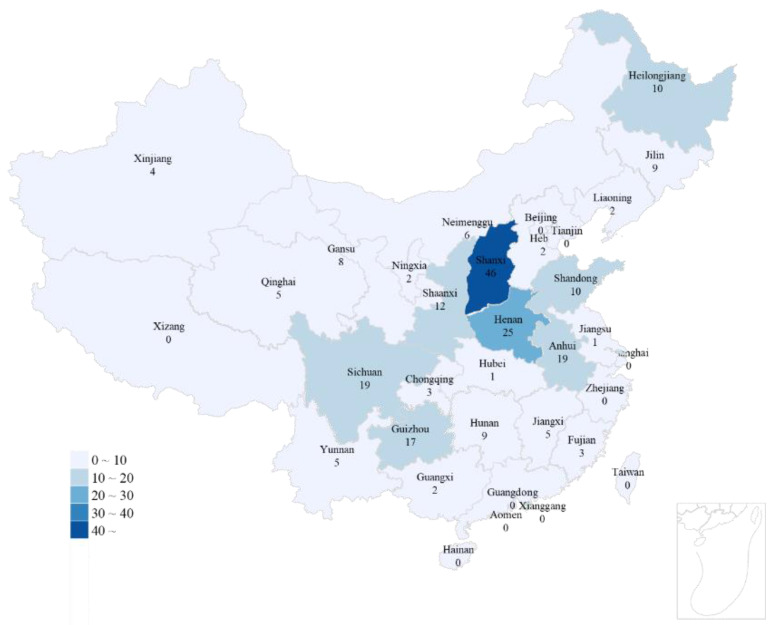
Accident quantity distribution map.

As shown in Figure 2, these 225 accidents cover 24 coal-producing provinces, which have geographical universality. Furthermore, these accident reports cover all types of accidents, except fire and blasting accidents, such as roofing and transportation accidents, which have universality of accident types. The accident data used in this study were obtained from the official websites of the State Administration of Work Safety of China, the State Emergency Management Administration of China, and the safety supervision administration of each province and city in China, which are credible. The 227 coal mine construction standards involved in the establishment of the rule database are from the reliable National Standardization Administration of China.

### 2.2. Extraction of Risk Factors for Coal Mine Building Design Based on Word2Vec

In order to make the establishment of the standardized rule database more targeted, and the compliance inspection more focused and efficient, it is necessary to first extract the most relevant coal mine construction elements from previous accident reports [28,29,30]. The extraction steps are as follows:

First step: Preprocessing

The accident analysis report is usually detailed and includes the name and location of the mine involved, and the time of the accident. After 225 accident analysis reports are integrated into Python, pandas and numpy modules are used to remove stop words and non-text parts from the text of the accident analysis reports. Then the jieba toolkit is imported to segment the text to form the initial corpus [31].

Second step: Training

A Word2Vec model can be used to quickly generate word vectors from the words in a given corpus. The purpose of this experiment is to explore the design factors of coal mine buildings that are closely related to a certain type of accident, that is, to distinguish the accident causes by category, without considering each type of accident. It is more appropriate to calculate the cosine value between word vectors to calculate the correlation between accidents, so a CBOW model is selected in this experiment [32].

The original accident analysis report contains six types of accidents. The training focuses on these six accident types. The Word2Vec model included in Python’s GenSim toolkit is invoked, feeding the preprocessed corpus into the model. In this study, the CBOW model is used to extract architectural elements, the model parameters are set to sg = 0, and the window size is set to windows = 3.

After subscribing each accident report, it can be represented as a spatial vector with fixed dimensions. For example, if there is an accident analysis report word vector ***A*** = (*x*_1_, *x*_2_, …, *x_n_*) an accident type word vector ***B*** = (*y*_1_, *y*_2_, …, *y_n_*), and the angle between the two is α,

(1) $$\cos\alpha =\frac{A\cdot B}{\left|A\right|\cdot \left|B\right|}=\frac{\sum_{i=1}^{n}x_{i}y_{i}}{\sqrt{\sum_{i=1}^{n}{\left(x_{i}\right)}^{2}}\sqrt{\sum_{i=1}^{n}{\left(y_{i}\right)}^{2}}}$$

then the closer cosα is to 1, the closer α is to 0, and the more similar the two word vectors are.

Third step: Clustering

Depending on the type of incident, the focus of compliance inspections varies. Using various types of accidents as search words, the five building elements with the most similarities to various types of accidents in the past are obtained, meaning a subsequent rule base can be established as the focus. Taking the roof accident as an example, the five architectural design risk factors most related to roof accidents are working surface, harvesting surface, safety pillar, ventilation roadway, and repairing roadway. Therefore, to realize the pre-control of roof accidents, these five architectural design risk factors in the coal mine architectural model are emphatically examined. The obtained results are shown in Table 1.

## 3. Establishment of the SQL Rule Database

### 3.1. Standardization of Building Information

To realize the compliance inspection of architectural risk factors, rules should be organized and standardized. Design specification information standardization is the basis for automatic inspection. In the past, scholars have developed different classification methods for different kinds of construction standards [33]. According to the purposes and requirements of inspection, Solihin and Eastman [34] roughly divided the rules into the following seven categories: (1) format specification of an architectural model; (2) specification for clear definition parameters; (3) special specification for specific building projects, such as mines and hospital construction projects; (4) design implementable related specifications, such as temporary support and templates; (5) specification for construction procedures; (6) warranty or maintenance issues related specification; and (7) relevant specifications of integrity of construction models. The building rules database covered in this study mainly refers to the specifications of the definition of parameters and design implementability.

Rule information standardization can be divided into three steps based on the relevant specifications of the building design risk factors in the work surface, as an example.

The first step is required to retrieve, classify, and filter the relevant specification terms. First of all, the specification clauses related to the construction factor of the working face output in the previous step are retrieved and the related clauses are summarized into a rule set for further classification. Then, the specifications in the collections of the previous step are classified. Among them, some norms are descriptive, qualitative requirements for components, or specifications to the sequence of construction processes, such as “the air volume required for the tunneling working surface should be consistent with the gun smoke can be discharged within 15 min after the gun”. Others are specifications that are quantified to a component, such as “30–50 m of the working surface pre-grouting”, including a quantitative property value, which can be determined by evaluating whether it is complied with. According to the above classification method, the rules extracted in the previous step are classified and divided into description rules and judgment rules. Finally, the rules that automatically check the BIM platform should comply with characteristics such as integrity, specificity, consistency, and availability. This process filters the description rules and leaves the judgment rules that can be checked.

The second step is to extract all constraint objects for each specification and organize the attribute constraints and parameter constraints for an object. The object is a specific building component in each provision. A building component is constrained by the building elements, and the attribute parameters are constraints on the components. Some judgment rules are directly constrained by the properties of a single component, and others are constraints requiring that a component is constrained relative to another component. These two rules are very different in the construction rules to check the plug-in, so they must regulate specifications. Concentrated normative entry is performed for further analysis. This article refers to the study of Chen et al. [35] to summarize the standard centralized judgment rules into eight types. The eight types of judgment rules are shown in Table 2.

The third step is to establish a standardized expression framework for the rules. The standardization framework starts from the risky building elements and then finds the sources of relevant coal mine design standards and design specifications that constrain the building risk elements. Specific normative clauses are analyzed to obtain constraint objects and constraints in the clauses, that is, building component names, attribute parameter constraints, constraint degrees, and so on. The final form of building rules into a standardized framework is shown in Table 3, where the degree word is the strength of the rule constraint.

### 3.2. SQL Rule Database Establishment

**Figure 3:**
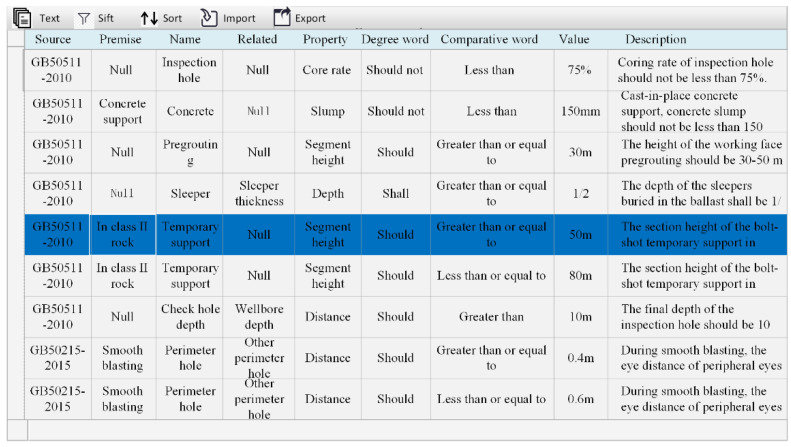
Rules database detail diagram.

To realize BIM platform access to the rule database, the rule database should have powerful storage, retrieval and interaction functions. MySQL is a small, open-source relational database management system. Due to its small size and high speed, it is suitable for the establishment of small databases, and it has a considerable number of interfaces that can interact with a BIM platform. Navicat for MySQL is a tool for managing and developing using MySQL. This research uses Navicat 15 for MySQL to build the risk rule base. First, the rule normalization table is established in Excel and then the MySQL database is imported to establish the rule base. The fields in the rules database based on accidents should correspond to the fields of the standardization framework. The specific situation of the database is shown in Figure 3. The field type selects more compatibility “varchar”. In addition, to ensure the effectiveness of the inspection, other than the “prerequisite” and “related components” fields, the fields are not allowed to be empty.

## 4. Development of Compliance Inspection System Based on BIM

### 4.1. Compliance Inspection Reasoning Mechanism Design

The core of the compliance inspection system is model inspection. BIM-based model checking can be seen as a matching command issued by BIM to match engineering data with rule data and output building components that do not comply with building design-related codes and have safety hazards. The BIM model can be regarded as a database containing all kinds of data of the model. To match the specific data in two databases with huge data volume, a complete and mature reasoning mechanism is required.

Reasoning refers to the process of inferring new conclusions from one or several existing premises or facts. Reasoning methods can be divided into three methods: forward reasoning, reverse reasoning and mixed reasoning. Forward reasoning is reasoning from existing and actual conditions to a conclusion, which can also be called factual reasoning. This study adopts a forward reasoning method, that is, starting from the building components that exist in the building model to trigger the corresponding norms or standards in the rule database and outputting an inspection report when all relevant rules are checked. The inspection report contains the names of non-compliant components, their properties, and the degrees of noncompliance. The criteria for judging the degree of noncompliance are shown in Table 4.

The specific reasoning steps are as follows:

(1) Select components that need to be checked for compliance in the inspection system interface.
(2) Query all relevant rules in the rule library according to the components selected.
(3) Select the environment or prerequisite for the components.
(4) Select the component properties of the inspection. If the property is a single component, check whether the attribute parameters of the component are complied with. If the selected attribute is determined using two or more components, such as the distance between components, the first component is selected as the center, a coordinate axis is established, the distance between the related components is measured, and then compliance is evaluated.
(5) Noncompliance declaration and output of inspection report.

**Figure 4:**
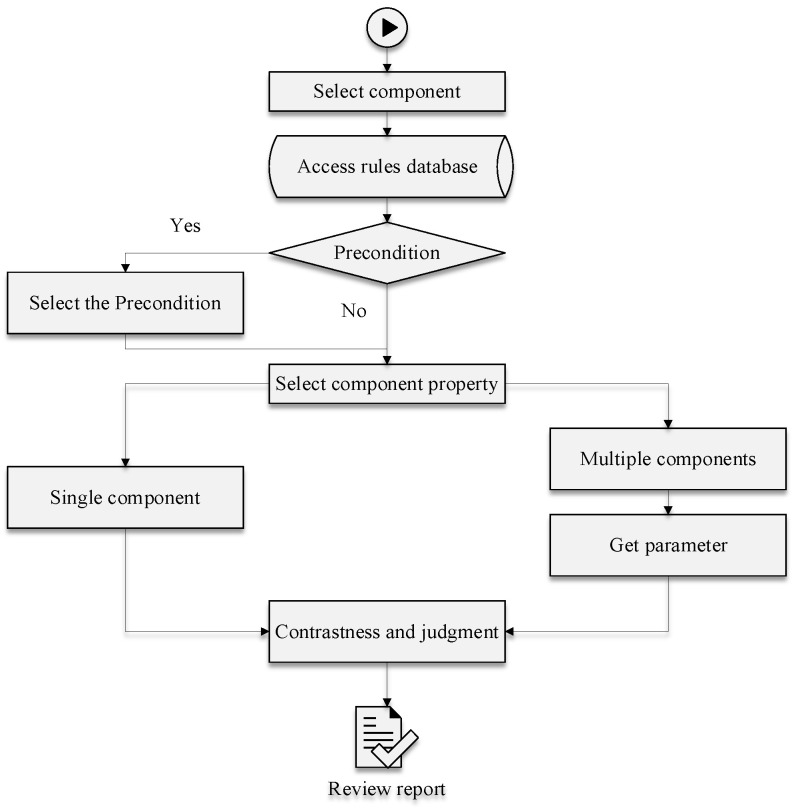
Reasoning process.

The reasoning process is shown in Figure 4.

### 4.2. Compliance Inspection System Creation and Testing

#### 4.2.1. Development Software Selection and System Construction Process

There are dozens of BIM software programs in common use today. Compared with other software, Revit has a higher usage rate in China and is more closely integrated with construction projects. The user interface is also friendly to nonarchitecture users and supports collaborative design. Therefore, this study uses Revit 2021 to develop the compliance inspection system.

Revit secondary development means that users can use any .NET compatible language to program through the API interface provided by Revit to integrate functions required by users into the Revit platform. Languages compatible with .NET include C#, C++, F#, JavaScript, and Visual Basic. NET, among others. Visual Basic .NET belongs to the Basic language. The syntax is similar to natural language and is mostly expressed in English. It is simple and easy to learn, and the existing Visual Studio platform supports writing in multiple languages. Consequently, Visual Studio 2022 is used for Revit code development.

**Figure 5:**
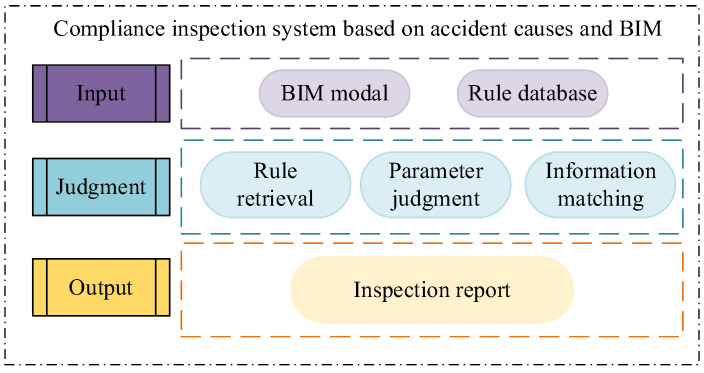
Overall system model diagram.

The establishment of the system is mainly divided into three parts: the input module, judgment module, and output module. An overall framework diagram of the system is shown in Figure 5.

The main function of the input module is to input rules. The rule database in this study uses an external database connected with the MySQL database through the Revit interface to access the rule base. The rule input can be manually added directly to the database or via batch input by creating an Excel table. The rule base can be continuously updated without changing the number of keys in the database.

The judgment module is the core part of the system, and its function is to evaluate whether the relevant parameters of the components in the BIM model meet the requirements of the relevant specifications. To make the system complete the compliance check of the components, it is necessary to realize three aspects: the retrieval of rules, the matching of engineering information and rule information, and the judgment of parameters. This study starts from a certain type of component rather than a specific instance. One inspection checks the compliance of the same type of component, which is efficient and can realize the function of batch inspection.

The role of the output module is to output the inspection result report. The inspection result is finally presented in the form of a pop-up window, which includes the component name, the ID of the component in the model, the component attribute, whether it is compliant, the degree of noncompliance, and a detailed description.

#### 4.2.2. System Test

(1) Test model testing

In testing the system, the first choice was to build a BIM test case. The test cases were built according to the relevant standard GB50511-2010_Code of Construction for Coal Mine Shaft Engineering. GB50511-2010 was chosen because it is widely used in China. The design information was added into the BIM model one by one, and the system test results were as follows: the number of non-compliant components detected accounted for 92.82% of the total number of non-compliant components that should be detected, of which the correct detection rate was 97.68. The specific results are shown in Table 5. These high-performance results show that the coal mine construction compliance inspection system constructed in this study is effective in detecting non-compliant designs in the model.

In addition, an error analysis was conducted to identify the source of the non-compliance detection errors. The analysis revealed that the non-compliance detection errors stemmed from errors in the cross-referencing of rule specific information with model component information, and that, as the naming of specific components in BIM model building is variable and prone to errors at the matching stage, it was not an error in the extraction and conversion of rule information and BIM information or an error in the logic of reasoning. This suggests that the use of compliance inspection systems requires an increase in the standardization of naming.

(2) Instance model testing

In order to test the effectiveness and adaptability of the system, the BIM model of the Balasu coal mine was selected as an example for testing. The Balasu Coal Mine is located 40 km west of Yulin City, Guangxi Province, China. The well field is approximately 13.4 km wide from east to west and 22.5 km long from north to south, with a total area of approximately 300.49 square kilometers.

**Figure 6:**
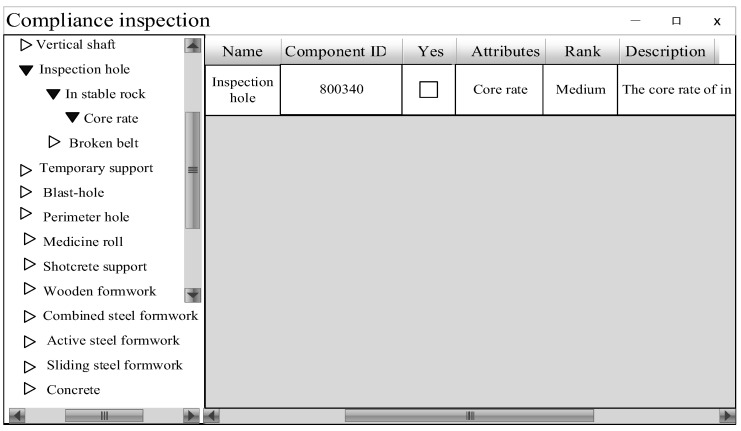
Inspection report.

The results showed that the model had an inconsistent component design, as shown in Figure 6. The coring rate of the inspection holes did not meet the specification requirements. After entering the ID in the model to find the component, the specific reasons for non-compliance are as follows: The coal mine is in a stable rock formation, and the coring rate of the inspection hole should not be less than 75%. However, in the BIM model of the Balasu Coal Mine, the coring rate of the inspection hole with ID 800340 is only 59%, so this inspection hole does not meet the requirements of the “Code for Construction of Coal Mine Roadway Engineering”. The inspection time for a single component of the model is only 0.23 s, which is a significant reduction compared with previous manual inspections.

#### 4.2.3. System Assessment

Most of the previous research on the accident-based identification of potential safety hazards in coal mines is based on the analysis of the causative mechanism of accidents. The purpose of these studies is to explain the mechanisms by which accidents occur, thereby providing a basis for risk assessment. However, it does not start from a specific physical model to identify and control coal mine safety risks. For example, Zhu [36] used the system theory accident causation model to analyze a large gas explosion accident, and found that the main reason for the accident was that there were many errors in organizational control, and then put forward corresponding suggestions for the prevention of gas explosion accidents. As the carrier of engineering data, BIM is mostly used in the coal mining industry to improve the design quality, and BIM is rarely used to identify potential safety hazards. For example, He [37] developed a BIM-based three-dimensional design system for coal mine roadways, which conveniently realized the three-dimensional design and visualization of coal mines. The superiority of the compliance inspection system established in this paper is reflected in the following. (i) The focus of inspection is determined by extracting coal mine building components with high potential hazards, which improves the extensive inspection approach of traditional compliance inspection. The compliance inspection is based on the cause of the accident, which makes the inspection focused and biased. (ii) The traditional compliance inspection is mostly manual, which leads to a large amount of time required for model inspection. The inspection time for a single component of this system is only 0.23 s, which improves the efficiency of inspection. (iii) The effective combination with BIM guarantees the correct inspection rate, and the inspection results are more convincing compared with the more subjective manual inspection. However, due to the subjective and variable nature of naming specific components in the BIM model building process, errors in the matching stage still exist. Therefore, the application of compliance inspection system requires the improvement of naming normality.

## 5. Conclusions and Outlook

Differing from a traditional design compliance check system, this research focuses on the discovery of accident causes according to the deep-level features of words, and combines them with the actual model to carry out research on the construction of a compliance check system. First, Word2Vec was used to train the word vectors from 225 coal mine accident analysis reports, and the building elements most related to roof accidents, water penetration accidents, and fire accidents were extracted. Then, the 227 current codes related to coal mine construction were searched with the subject of risky coal mine construction design elements, and the rule set obtained after the search was standardized to establish a rule base for risks. Finally, the risk rule base was linked with the BIM platform for development, and a focused and efficient automatic inspection system for coal mine construction compliance was developed based on the accident causes and the BIM platform.

Theoretically, this study applies the secondary development of a BIM platform to the field of coal mine construction and provides an idea from the design stage for the pre-control of coal mine accidents. Accidents caused by coal mine building design are often significant and destructive. By checking for non-compliant designs through the compliance check system and modifying or rebuilding the checked non-compliant designs, accidents during subsequent construction and mining can be effectively reduced.

Practically, this inspection system is of great value. Firstly, the study begins with accidents that have already occurred, which makes the compliance inspection more targeted. Secondly, when the system was tested, the detectable rate of non-compliant components reached 92.82%. The system test verifies the validity of the check logic, and the system can effectively improve the quality of the compliance check. With a single component check time of only 0.23 s during system testing, the system is able to shorten the time taken for model checking, which in turn improves the efficiency of model checking. Thirdly, non-compliant designs that are checked are corrected in a timely manner to ensure correct construction, avoid accidents arising from the building design during subsequent construction and mining, and improve the safety of coal mine buildings. Furthermore, the establishment of the standardization framework of the rule database makes it possible to replicate the compliance inspection system in the design of coal mines in other geographical environments. By establishing a standardized rule framework and integrating the norms into the rule database, the localization of the compliance inspection system can be realized. Finally, the BIM platform itself is used to develop the compliance inspection system, which makes installation and inspection operations easier and facilitates real-time inspection during the design process.

The focus of the research is the extraction of construction causative factors of coal mine accidents and the design of a BIM-based compliance inspection system. However, in terms of how to realize the pre-control of coal mine accidents through the non-compliant design detected by the compliance automatic inspection system, only a preliminary discussion has been carried out, and further research is needed in the future. In addition, due to the limitations of data and time, the compliance check system has not been checked for its applicability to different countries or regions.

## Figures and Tables

**Table 1:** Key coal mine building elements and similarity.

Accident Type	Risk Factors for Coal Mine Building Design				
Roof	Working surface (0.9788)	Stoping workface (0.9778)	Coal rock pillar (0.9771)	Return laneway (0.9680)	Repairing roadway (0.9450)
Flood	coal rock pillar (0.9859)	Water detection points (0.9744)	Drainage system (0.9699)	Internal warehouse (0.9465)	Water inflow (0.9372)
Gas	Return laneway (0.9723)	Ventilation system (0.9709)	Coal rock pillar (0.9690)	Working surface (0.9655)	Stoping workface (0.9480)
Transport	Transporter (0.9778)	Buffer (0.9773)	Power system (0.9754)	Strand (0.9659)	Point pillar (0.9631)
Electromechanical	Power system (0.9728)	Filter (0.9701)	Transporter (0.9683)	Sensor (0.9624)	Ventilator (0.9598)
Other	Overhead lines (0.5782)	Ventilator (0.5695)	Tape dispenser (0.5688)	Intake airway (0.5656)	Steel strand (0.5652)

**Table 2:** Judgment rule types.

Number	Type Name	Example
1	One-to-one constraints on component properties without preconditions	Coring rate of inspection hole should not be less than 75%
2	One-to-one constraints on component properties with preconditions	Cast-in-place concrete support, concrete slump should not be less than 150 mm
3	One-to-many constraints on component properties without preconditions	The height of the working surface pre-grouting should be 30–50 m
4	One-to-many constraints on component properties with preconditions	The height of the temporary anchor spray support in Class II rock formation should be 50–80 m
5	A single property constraint involving multiple components without preconditions	The final depth of the inspection hole should be 10 m greater than the design depth of the wellbore
6	A single property constraint involving multiple components with preconditions	When pouring tremie pipe, the distance between the pipe and the end face of the slot should be 1.5 m
7	Multi-attribute constraints involving multiple components without preconditions	The depth of the sleepers buried in the ballast shall be 1/2 to 2/3 of the thickness of the sleepers
8	Multi-attribute constraints involving multiple components with preconditions	During smooth blasting, the distance of surrounding apertures should be controlled within 0.4–0.6 m;

**Table 3:** Standardization framework for building rules.

Source	Premise	Name	Related	Property	Degree Word	Compara-tive Word	Value	Description
GB50511 -2010	Null	Inspection hole	Null	Core rate	Should not	Less than	75%	Coring rate of inspection hole should not be less than 75%.
GB50511 -2010	Concrete support	Concrete	Null	Slump	Should not	Less than	150 mm	Cast-in-place concrete support, concrete slump should not be less than 150 mm.
GB50511 -2010	Null	Pregrouting	Null	Height	Should	Greater than or equal to	30 m	The height of the working surface pregrouting should be 30–50 m.
GB50511 -2010	Null	Pregrouting	Null	Height	Should	Less than or equal to	50 m	The height of the working surface pregrouting should be 30–50 m.
GB50511 -2010	In class II rock	Temporary support	Null	Height	Should	Greater than or equal to	50 m	The height of the temporary anchor spray support in Class II rock formation should be 50–80 m.
GB50511 -2010	In class II rock	Temporary support	Null	Height	Should	Less than or equal to	80 m	The height of the temporary anchor spray support in Class II rock formation should be 50–80 m.
GB50511 -2010	Null	Inspection hole depth	Wellbore depth	Distance	Should	Greater than	10 m	The final depth of the inspection hole should be 10 m greater than the design depth of the wellbore.
GB50511 -2010	Tremie pipe	Blanking conduit	Slotted end	Distance	Should	Equal to	1.5 m	When pouring tremie pipe, the distance between the pipe and the end face of the slot should be 1.5 m.
GB50511 -2010	Null	Sleeper	Sleeper thickness	Depth	Shall	Greater than or equal to	1/2	The depth of the sleepers buried in the ballast shall be 1/2 to 2/3 of the thickness of the sleepers.
GB50511 -2010	Null	Sleeper	Sleeper thickness	Depth	Shall	Less than or equal to	2/3	The depth of the sleepers buried in the ballast shall be 1/2 to 2/3 of the thickness of the sleepers.
GB50215-2015	Smooth blasting	Surrounding aperture	Other aperture	Distance	Should	Greater than or equal to	0.4 m	During smooth blasting, the distance of surrounding apertures should be controlled within 0.4–0.6 m;
GB50215-2015	Smooth blasting	Surrounding aperture	Other aperture	Distance	Should	Less than or equal to	0.6 m	During smooth blasting, the distance of surrounding apertures should be controlled within 0.4–0.6 m;

**Table 4:** Relationship between degree words and noncompliance degrees.

Degree of Noncompliance	Degree Words				
Serious	Ought not	Shall not	Forbidden	Must	Shall
Medium	Should	Should not			
General	Can				

**Table 5:** System test results.

Sections	Number of Components	Number of Detections	Correct Number of Checks
Construction preparation	4	4	4
Common method of construction of vertical shafts	77	73	71
Special method of vertical shaft construction	262	243	238
Extension and Rehabilitation of Vertical Shafts	6	5	5
Inclined shaft and flat cavern construction	6	6	5
Roadway construction	79	72	71
Concealed shafts and chambers	6	5	5
Ancillary work	108	99	98
Working Environment and Occupational Hazards	9	8	8

## Data Availability

The data presented in this study are available on request from the corresponding author. The data are not publicly available due to privacy restrictions.
